# Science Runs and the Debate Brakes: Somatic Gene-Editing as a New Tool for Gender-Specific Medicine in Alzheimer’s Disease

**DOI:** 10.3390/brainsci10070421

**Published:** 2020-07-02

**Authors:** Pamela Tozzo, Silvia Zullo, Luciana Caenazzo

**Affiliations:** 1 Department of Molecular Medicine, Laboratory of Forensic Genetics, University of Padova, Via Falloppio 50, 35121 Padova, Italy; luciana.caenazzo@unipd.it; 2 Department of Legal Studies, University of Bologna, 40121 Bologna, Italy; silvia.zullo@unibo.it

**Keywords:** Alzheimer’s disease, ethics, genders, health and new technologies, somatic gene-editing

## Abstract

Gender-specific medicine is a discipline that studies the influence of sex and gender on physiology, pathophysiology, and diseases. One example in light of how a genetic-based disease among other diseases, that impact on sex, can be represented by the risk of developing dementia or Alzheimer’s disease. The question that comes into focus is whether gene-editing can represent a new line of investigation to be explored in the development of personalized, gender-specific medicine that guarantees gender equity in health policies. This article aims to discuss the relevance of adopting a gender-specific focus on gene-editing research, considered as a way of contributing to the advance of medicine’s understanding, treatment, and prevention of dementia, particularly Alzheimer’s disease. The development or improvement of cures could take advantage of the knowledge of the gender diversity in order to ascertain and develop differential interventions also at the genetic level between women and men, and this deserves special attention and deep ethical reflection.

## 1. Introduction

Historically, medical research has mainly been conducted using the male body as the norm, as the basis of clinical studies. Women did not participate in clinical trials and even when they did, the data was not desegregated by sex. In the last decades, medicine has started to recognize the importance of taking sex into account as there is increasing evidence that symptoms and responses to medical treatment may be very different between sexes [1,2]. Medicine began to understand that women are not small men and that harms can occur when sex and gender are not considered. Extrapolating conclusions of research conducted with men and applying them to women can have detrimental consequences related to diagnoses, treatment, and prevention. Gender-specific medicine, as a new medicine, has made significant progress over the last couple of decades and demonstrated to have a good potential to transform not only medicine but also global healthcare. It studies the influence of sex and gender on physiology, pathophysiology, and diseases. It embraces differences between males and females pertaining to both psychological and social aspects and is important in improving our knowledge in all aspects of human health dealing with sex and gender differences: this discipline finds its application from public health perspectives to clinical practice, from research in basic sciences to the detection of diagnostic gender-specific markers [2]. 

Originally the main differences between men and women detected and studied by gender medicine were regarding the immune system and medical conditions such as hypertension and heart diseases. Women experience more often autoimmune diseases and suffer more frequently from pain and neurodegenerative changes. Men have shorter life expectancy but relatively more healthy years of life, which is mostly ascribed to psychosocial determinants. 

Current clinical medicine addresses individual risk factors based on sex and gender-sensitive health programs in order to improve the health-related quality of life for men and women. A great deal of research has led to a growing awareness of differences in physiology and pathology between men and women throughout the entire life course, following the well-known concept regarding the distinction between the terms sex and gender. To distinguish sex and gender, different definitions have been reported in the literature, which we could summarize as follows: “Sex” is a biological construct and refers to a set of biological attributes includes hormone function, reproductive/sexual anatomy chromosomes, cells, and tissues or reproductive differences between males and females. “Gender” refers to the socially constructed roles and behaviors considered appropriate for men and women, so it refers more strictly to psychological aspects, social and cultural rights, involving the circumstances in a person’s life, the belief that each person has of his/herself and the behavior that men and women have in the social sphere, how they appear, how they think, what they feel, how they dress and how they perceive the world in which they live [3,4,5,6,7,8]. Gender regards, in other words, the cultural and symbolic representation of femininity and masculinity in society.

A set of different concepts have been used to describe the same idea, that is the idea that gender should also be a determinant of health and that both clinical practice and health policies should take this into account: gender-specific medicine; gender medicine; gender-based medicine. Although these terminologies use the word “gender”, all aim to encompass both sex and gender differences. However, more recently, as a result of the contribution of other scientific areas, namely social sciences, where gender issues are being discussed since the 70s, some authors have advanced the need to clarify these concepts and to use them in a precise and standardized way in the context of medicine. Sex and gender are sometimes used interchangeably, and when discussing sex differences (genetic, biological, and phenotypic) the word gender is used. However, sex refers to a personal biological status, based on a combination of anatomy, genetics, and hormones, it is a biological construct. Whereas gender is a social construct, it refers to attitudes and behaviors socially and culturally constructed and expected from women and men; it refers to the broader social, cultural, and normative facts that affect health. WHO has defined gender as one of the main social determinants of health, recognizing the weight of gender inequalities [9].

Moreover, it is widely demonstrated in the scientific literature that women live longer than men, and this difference in life expectancy is a worldwide phenomenon indicating that human longevity seems strongly influenced by sex and gender as a combination of biological sexual characteristics (anatomy, reproductive functions, sex hormones, expression of genes on the X or Y chromosome) and factors related to behavior, social role, lifestyle, and life experiences. The epidemiology of age-related diseases is substantially different between genders and changes dramatically in women after menopause. Women die at higher rates than men of chronic lower respiratory diseases, cerebrovascular diseases, Alzheimer’s disease (AD), influenza and pneumonia, septicemia, and hypertension-related diseases [10]. 

An analytical approach that integrates a sex and gender perspective into the development of health research, policies, and programs, as well as health planning and decision-making processes has been defined in the literature as Sex- and Gender-Based medicine [11]. Sex and gender are not mutually exclusive because they are integrally related and influence health in different ways [12].

The current ability to manipulate any genomic sequence by gene-editing has created different opportunities in order to treat many different diseases and disorders. In this context, the increasing body of sex and gender-specific evidence, at different levels, may lead to a change in how medical research and education approaches teach both women’s and men’s health.

Specifically, the use of human genome editing to act on somatic cells for purposes of treating genetically inherited diseases is already in clinical trials. Somatic cells contribute to the various tissues of the body but not to the germline, meaning that, in contrast with heritable germline editing, the effects of changes made to somatic cells are limited to the treated individual and would not be inherited by future generations [13].

Gene-editing technologies have been around for over a decade. Zinc finger nucleases (ZFNs) and transcription activator-like effector nucleases (TALENs), two gene-editing technologies, were discovered in 2005 and 2010 respectively [14]. Gene-editing using designer nucleases such as ZFNs, TALENs, and Cas9 (Crispr-associated protein 9) RNA-guided nuclease (or Crispr [clustered regularly interspersed short palindromic repeats]/Cas9) are revolutionizing genetic engineering in vitro and in vivo. RNA tool (Crispr) functions as a guide for Cas proteins to target specific parts of the genome, which are subsequently cut by Cas proteins. These cut strands can be exploited to modify the DNA nucleotide sequence and insert genes at the cut site. ZENs and TALENs are relatively precise techniques, but have the disadvantage that they need engineered proteins to target specific sequences of DNA, a procedure that requires time and resources. Crispr/Cas9 is now the nuclease of choice because it does not involve producing target-specific proteins and requires only adjustments on a short region of the single-guide RNA (sgRNA) to make it target specific. It is at least as efficient as the other methods and is less cytotoxic. We have reason to say that this advancement could provide important changes in our concept of gender-specific medicine in the genomic era, in particular in implementing the principles of gender-specific medicine to various diseases’ research [15]. 

This article aims to discuss the relevance of adopting a gender-specific focus on gene-editing research, considered as a way of contributing to the advance of medicine’s understanding, treatment, and prevention of dementia, particularly Alzheimer’s disease (AD). It is in our opinion a socially valuable topic of analysis, given the burden of AD and dementia in modern societies, which targets more women than men, and also the need for medicine to acknowledge the biological and social differences between men and women. However, gene modification is also an issue that raises ethical concerns about the limits of biomedical research. 

## 2. Somatic Gene-Editing for Developing a Patient-Specific Therapeutic Strategy

In light of the foregoing remarks, it seems clear that the opportunity to intervene at the genome level by correcting an individual’s genetic makeup may be used to revert an underlying genetic mutation to a variant not associated with disease or engineer a cell so that its phenotype differs from that of a normal cell could result better able to resist or prevent disease altering a trait not associated with disease.

These approaches could be applied to treat or prevent a disease and, where necessary, they may also consider how the interventions modifying the genome should include not only sex but also gender as a crucially important variable on the risk of diseases and in the experimental protocols.

Genome editing is being developed to treat not only monogenic diseases but also infectious ones and diseases that have both a genetic and an environmental component. As Doudna writes in his work entitled A Crack in Creation: “Because Crispr allows precise and relatively straightforward DNA editing, it has transformed every genetic disease—at least, every disease for which we know the underlying mutation(s)—into a potentially treatable target” [16].

New gene-editing tools like Crispr-Cas9 enable precision genome engineering in four areas: (a) increase DNA cutting precision; (b) increase on-target knock-in of sequence variants and transgenes; (c) increase transcriptional control of edited genes; (d) increase specificity in delivery to a specific cell or tissue. Design of next-generation gene and cell therapies will likely exploit a combination of these advances [17,18].

If we consider the four types of precision genome engineering listed above and the idea of making genetic changes to somatic cells, referred to gene therapy, we note, on the one side, that this idea is not new and considerable progress has been made over the past several decades toward clinical applications of gene therapy to treat diseases [19], but, on the other side, prospects for future applications of gene therapy have been greatly enhanced by improvements in genome-editing methods. For example, genome editing can be carried out ex vivo or in vivo. In ex vivo editing, it is possible to conduct a number of checks on the edited cells before they are administered to a patient because the cells are first manipulated in the laboratory, outside the body, but ex vivo editing is suitable only for certain cell types. By contrast, in vivo editing allows many types of cells and tissues to be edited, but poses additional safety and technical challenges because it involves performing genome-editing procedures directly into a patient’s body, in order to modify targeted cells. In vivo somatic gene-editing existing applications are aimed at improving strategies that specifically edit stem cells to regenerate tissues and correct disease-causing mutations. However, compared to ex vivo approaches, in vivo approaches pose greater challenges with respect to efficient delivery of the genome-editing to the right cells in the body, ensuring that the correct position in the genome has been successfully edited [20,21].

More specifically, these existing methods for delivering genome engineering in human-induced pluripotent stem cells (hiPSCs), which are a type of pluripotent stem cells that can be produced from adult cells by genetic reprograming, represent an opportunity to examine the contribution of pathogenic alleles to molecular and cellular phenotypes. However, the practical application of genome-editing approaches in hiPSCs has been challenging [22]. We know that combining this with recent advances in genome editing techniques, such as the Crispr system, has provided only an ability to repair putative causative alleles in patient lines. This has enabled the analysis of isogenic cells that differ in a single genetic change to assess the molecular and cellular phenotypes that result from this abnormality. These isogenic cell lines can be used not only to understand the cellular consequences of disease mutations but also to perform high genetic and pharmacological screens to both understand the underlying pathological mechanisms and develop novel therapeutic agents to prevent or treat such diseases.

So, using the latest Crispr-Cas9 technology, scientists believe it will be possible to introduce precise modifications into the genome of patient-derived hiPSCs. These modifications will “correct” the putative risk alleles present in patient lines by replacing them with wild-type alleles. Following genome editing, it would be possible to differentiate both unmodified and modified hiPSC lines in disease-relevant cell types. When derived from the same patient, these cell lines will have an identical genetic background, differing only by the presence or absence of a specific risk allele. These powerful models may be a valuable research tool and provide a novel treatment for single gene disorders, and in the longer term, could be an important tool in the fight against polygenic and infectious diseases. 

In the future, the hope posed on hiPSCs cells generated from genetic disease’s patients is promising because they may help us discover novel mechanisms of diseases. This could lead to the development of new and unprecedented drugs for patients, and in the case of known familial mutations, these cells could be targeted through the use of advanced gene-editing techniques in order to correct the mutation and be used for future cell transplantation therapies [15,23,24,25,26].

In this context of applications, it will be necessary to understand what transformations human gene-editing brings to the future of gender-specific research agenda in order to consider the impact of the genomic era on gender-specific medicine where the mechanisms of genetic and hormonal factors combine with other factors in giving rise to a different approach to intervention on the expression of genetic vulnerabilities, as well as to exploring the consequences of this new technology for men and for women in connection with a specific disease [27]. 

Nevertheless, this kind of studies need to be developed and differentiated in relation to sex and gender differences in order to obtain a valid result, providing better control cell lines for comparisons and potentially better phenotypes, respectively. Furthermore, it will be relevant to consider gender differences that affect the way man and women respond to therapies and treatments, and how to be treated relatively to their care as for example in clinical trials or healthcare programs.

But there are several ethical challenges that need to be addressed in working toward such a goal.

## 3. Somatic Gene-Editing and Gender-Specific Medicine: Addressing the Ethical Issues

As just noted, the research previously described cannot be advanced without taking account of the ethical concerns it raises [28,29]. 

A promising work has been published on two mice models of Alzheimer’s disease in which CRISPR-Cas9 nano-complexes were used, with few secondary effects, showing they were effective in the adult mouse brain, but without any preliminary considerations on the impact of gender on this kind of research [30]. The possibility of using gene-editing techniques on animal models to control the evolution of neurodegenerative diseases is extremely important from the point of view of scientific progress but also requires an awareness of potential and risks not only from the scientific community but also from the public opinion. Nevertheless, we believe that the development or improvement of cures could take advantage of the knowledge of the gender diversity in order to ascertain and develop differential interventions also at the genetic level between women and men, and this deserves special attention and deep ethical reflection.

A particular concern is about the potential for the implementation of genome editing techniques in certain contexts of biomedicine to have an impact on a fair distribution of advantages or opportunities among different groups in a society [31], as for example in Gender-Specific Medicine. This concern requires us to attend to the gender-specific needs to ensure that the application of this new biotechnology may affect welfare without unfair discrimination among people. 

The key objective on a local and global scale in biomedicine research, application, and management is not gender equality per se, but the recognition of the different needs of men’s and women’s health by virtue of their differences. Biological differences cannot be removed, but their potentially harmful effects, for one group over the other, can be prevented with health policies that properly take them into account, with a focus on equity rather than equality. Starting from this condition of equality in gender diversity, a strategy to be applied is to ensure that both men and women have opportunities to maximize their health potential, with respect to their biological diversity, taking into account different health needs [3].

According to what reported in 2016 by a Nuffield Council on Bioethics document [14], one focus on responsibility for producing and addressing injustice is the extent to which differences are intrinsic or socially constructed: while there is no question that women experience injustice, harm, and indignity in all societies. The forms of this unfair treatment can be highly cultural, social, and historical. The features of any society are complex, interdependent, and dynamic, but public policy measures often imply and express consistent common values and may be articulated around a collective vision of the desirable future state that they are expected to contribute to bringing about. How genome technologies are considered in the society can both betoken and consolidate essential features of society by posing important questions about what is for individuals or for society to determine, how common challenges are met and how goods are distributed [32]. 

In this view, genome editing should take into account gender determinants because, even if it regards the genetic make-up, it is not neutral with respect to achieve outcomes of care on man and women, as will be further discussed. In fact, various aspects of genetic risk of having a particular allele vary by gender, so we have a strong ethical reason to take this into account when considering the development of new gene-editing therapies.

In cases where there are sex and gender differences in health determinants, then any application of this new technology that does not take into account these differences would be unfair. 

More specifically, to assess somatic cell genome-editing applications, ethical concerns are focused on benefits and risks within the existing regulatory system of ethical norms that have allowed the current research and clinical meeting and development of somatic cell and gene therapy. 

This regulatory framework includes a wide range of preclinical test and study designs to support the clinical development of therapies based on edited cells and these studies have to be compliant with international ethical guidelines for biomedical research involving human subjects. This aspect is of great relevance because human genome editing is cheap and easier to perform so it may be somewhat more difficult to control than traditional gene therapy, but the cellular manipulations and delivery of edited cells to the patient continue to require high quality and high ethical standard ground. 

The discovery of hiPSCs cells is ground-breaking, as it means that patient-specific cell lines can be established easily, and this fact would make it possible to create cell lines that are genetically tailored to a patient. The intent of each of such modifications could be to treat or prevent disease but also to modify phenotypic traits in the treated cells or tissues. This regenerative medicine, including the testing of transplantation of cells into live tissues and organs, is moving forward for disease models on rodents for late-onset genetic-based diseases, such as neural progenitor cells and mesenchymal stem cells, but remains restricted because of ethical concerns in accepting this medical practice and because of the safety and efficacy issues that need to be addressed as part of this process [33].

Lastly, from the ethical perspective, we claim that if men and women respond identically to therapy (in this case somatic gene cell therapy), the issue of taking into account gender-specific medicine in clinical trials would be less important. However, due to the fact that current knowledge on this crucial point is incomplete, we believe it would be relevant to take into separate account man and women in the genome editing research for having sufficient information to make informed evaluations about gender-specific treatment and, for example, about the use of hiPSCs in drug-related research. Biologic differences between men and women may reflect genetic, physiologic, lifestyle, cultural, and social differences, although the mechanisms explaining these differences are still unknown to a great extent. In addition, differences between gender responses to drugs and other medical interventions are now being reported more frequently, but the results of medical research have been often generalized, without having sufficient evidence that these results would apply to women. 

In the field of dementia research, the question is particularly acute: for example, women are at greater risk of developing Alzheimer’s disease, whereas men are at greater risk of developing vascular dementia and, here, gender comes into play when societal factors create opportunities for advanced education and healthy lifestyles [34]. Therefore, sex and gender differences in the development of dementia highlight factors that require further investigation in order to use biological knowledge and other social health information to predict individual disease risk, identify disease subcategories, determine which individuals are most likely to respond to therapy, and provide personalized treatment and social assistance.

## 4. Alzheimer’s Disease: An Example of a Possible Application of Gender-Specific Somatic Gene-Editing

In the effort to understand the risk of developing dementia or Alzheimer’s disease, among other diseases, increasing attention has been paid, in recent years, to the differences between men and women in the causes and manifestations of neurological diseases, as well as, to the response to treatment and to outcomes. 

The lens of sex and gender that underpins gender-specific medicine are likely to transform medical and research approach to identify risk factors for dementia or Alzheimer’s disease. The societal impact of Dementia is of paramount interest nowadays, since it is a common age-related disease and population is ageing more and more in Western Countries. Epidemiological researches have demonstrated that women are at higher risk than men for developing dementia or AD: reasons for these differences are not completely known and it is still debated [35]. The analysis of Neu and collaborators has highlighted that there are not sex-related differences in risk of AD from 55 to 85 years of age: however, women have a greater risk than men between ages 65 and 75 [36].

It is well known in the literature that the strongest susceptibility variant for AD is the E4 allele of apolipoprotein E gene (APOE). APOE protein shows three major isoforms (APOE2, APOE3, and APOE4) that are respectively encoded by E2, E3, and E4 alleles. E4 allele is associated to a three- to four-fold probability of developing AD and also an earlier onset age. Furthermore, carriers of two E4 alleles have an even higher risk of AD than carriers of one allele [37]. The E2 allele, by contrast, has been demonstrated to have protective effect that is associated with longevity and a lower risk of AD [36].

The allelic variant APOE E4 is not only associated with higher risk of developing sporadic Alzheimer’s disease, but also in developing age-related cognitive decline: this last association has not been so deeply characterized as the one to AD, since questions remain about whether APOE E4 effects on cognitive decline are similar in men and women [38,39,40].

The APOE E4 allele effect in women by comparison with men represents a good example of a biological factor as a genetic variant that interacts with other biological factors, as hormones, or other genes hosted on chromosome X or Y, or with gender-related factors such as education, physical activity, behavioral preferences, type of occupation [35]. When considering men and women carrying APOE E4 allele, it seems that women have a greater risk of developing Alzheimer’s disease than men with the same genotype. This sex-based difference related to the presence of APOE E4 allele has relevant consequences in treatment trials, diagnostics, and therapeutics. Relatively to mild cognitive impairment (MCI), even if it is well known that APOE E4 allele is associated with a greater risk to develop this disease, it is debated in Literature if sex determinants influence the transition from MCI to AD or dementia in APOE E4 carriers. Some studies have shown that women expressing the APOE E3/E4 genotype have an increased risk of MCI between the ages 55 and 70 [36], between ages 70–80 [41] and of AD between the ages of 65 and 75 [36]. Lehmann et al. in 2006 described a correlation between gender and episodic memory impairment in E3/E4 carriers: women were affected by worse impairment than men in the ages of 70–74 [42]. This means that carriers of APOE E4 alleles could benefit from early treatments for MCI and AD, taking into consideration the gender differences emerging from epidemiological studies [36].

The Lancet Neurology Commission has just discussed the increasing costs of AD and associated dementias, suggesting actions for an innovative research approach in developing prevention and treatment strategies [43]. What is relevant in this analysis is the commission’s emphasis on the need for new approaches to diagnose and treat AD in the context of new cost-benefit analysis models by which to optimize the use of resources and improve quality of life for patients affected by Alzheimer’s disease. The commission also seems to recommend a particular focus on the effect of sex on AD and other dementias essential in advancing our understanding, treatment, and prevention of these disorders. The commission recognized that AD and other dementias disproportionately affect women and underlined the relevance of empirical data about sex differences and emerging sex-specific findings in dementias in order to assess the scientific approach to these illnesses for the benefit of both women and men.

In the matter of treatment, the commission found that sex-specific genetic and hormonal factors, as just mentioned, might contribute to variance in clinical efficacy. In addition to differences in genetic or brain-based vulnerabilities, societal factors also play a role in the risk of dementias and their outcome. A wide range of behavioral and lifestyle choices also affect risk factors and the degree of disability in individuals with dementia: An individual’s behaviors and experiences over his/her lifespan affect the brain, and many of these factors vary by sex. Sex differences, extending from genetic to psychosocial domains, are relevant to productive research, and they are crucial for defining priorities for public health planning. The Lancet Neurology Commission thus provides an opportunity to develop the research agenda for AD and other dementias where women remain underrepresented in biomedical research.

In the case of APOE genes expression in dementias we have to consider that: the APOE2 is the allelic variant which seems to be more favorable, since it brings a lower-than-average risk of getting AD; APOE3 which is the most common form is associated with an average risk and APOE4 entails three to five times increase of the average risk of getting Alzheimer’s disease. CRISPR would ease genomic interventions in order to change one allelic form of the APOE gene into a more favorable allele, even if editing the germline to prevent such disorders seems highly complex, especially for genetic defects that present risks in combination with environmental factors or lifestyles, such as Alzheimer’s disease. 

As previously noted, sex and gender differences have been reported in the incidence and prevalence of dementia; however, the reasons for these differences are not yet clearly understood, and it will be necessary to explore and increase gender-specific medicine in this field. The question that comes into focus is whether gene-editing can represent a new line of investigation to be explored in the development of personalized, gender-specific medicine that guarantees gender equity in health policies. In fact, a better understanding of the biology of sex differences in cognitive function will not only provide insight into AD prevention, but is also integral to such prevention. Of course, gene-editing cannot remove biological differences, but its potentially harmful effects, on one group relative to another, can be prevented with a research strategy that properly takes them into account promoting equity between genders [3]. The ongoing debate about the need for medicine to develop a sex and gender perspective, in order to understand how biological sex and the sociocultural aspects of gender affect health and disease for men and women, should not be crushed and reduced by the traditional ethical issues that plague the field of biomedical research.

## 5. Conclusions

Today, we move with unexpected rapidity into the exploration and manipulation of the genetic code, with the newest and arguably most effective genetic engineering tools such as Crispr-Cas9. The opportunity to intervene at the level of the genome by correcting a person’s genetic makeup may be helpful to understand how the interventions that modify the genome should consider gender as a crucially important health determinant, and in this direction, gene-editing could and should address gender differences not to achieve equality per se, but to meet the needs of individuals of different genders.

If we recognize that AD affects more women than men, it is fair to say that more women than men would benefit from gene therapy targeting AD. This can be seen from the point of view of the benefits for women, but also of the risks because if these procedures have adverse effects, they will fall more on women who disproportionately suffer from some diseases compared to men and who are also more exposed to social vulnerabilities and inequality.

Devoting attention to gender differences should become standard practice in health policy, as by opening new perspectives in terms of the appropriateness, effectiveness, and equity of prevention and care initiatives. It affects the quality and sustainability of providing health services by improving its results and cutting its costs.

Among these fields are those where the aim is to improve our knowledge in all aspects of human health that deal with the differences between men and women: from research to the detection of diagnostic gender-specific markers and from public health perspectives to clinical practice. In fact, the development or improvement of cures could take advantage of the knowledge of gender diversity in order to ascertain and develop differential interventions also at the genetic level between women and men. For example, instead of treating Alzheimer’s disease as a homogeneous disease, genetics and other diagnostic methods hold the potential to identify functional disease subtypes that could be specifically targeted, increasing diagnostic accuracy for Alzheimer’s disease patients. The potential role of gene-editing tools in advancing precision medicine for Alzheimer’s disease may not only improve accuracy of dementia diagnosis, thus enabling more personalized treatment strategies, but also speed up the discovery of new drugs and interventions.

If the technical feasibility and low cost of Crispr-Cas9 is likely to make this technology widely used, it is important to follow and test other hypotheses not only to eliminate single-gene disorders, insert protective genes, and potentially replace or modify genes to enhance physical and mental traits, but also manipulate genomic structure to test the impact of the intervention on the phenotype, in accordance with a gender-specific perspective. 

We think it would be a mistake to use Crispr-Cas9 and gene-editing technologies without paying attention to these aspects. Rather, we postulate that gender differences affect the relative weighting of potential promises and the real outcome of these technologies, as it is required by the main assumptions of personalized gender-specific medicine. 

We believe that the development or improvement of cures could take advantage of the knowledge of the sex and gender diversities in order to ascertain and develop differential interventions also at the genetic level between women and men, and this deserves special attention and deep ethical reflection, given the topicality of the subject and the impact of recent authoritative publications on the subject, which will certainly arouse interest in public opinion in the near future.

The applicability perspectives that gene-editing techniques disclose are enormous and, as with any type of new technology, are still difficult to predict. The ethical acceptability of each of these applications will go specifically evaluated into the future, “putting them to the test” of the current regulations, which, in this case, should prove inadequate should be optimized or, at least, rethought.
